# Supplementary material for: The genome sequence of the Golden Plusia, Polychrysia moneta (Fabricius, 1787) (Lepidoptera: Noctuidae)
Source: Wellcome Open Res. 2026 Jan 23;11:68. [Version 1] doi: 10.12688/wellcomeopenres.25811.1 (PMC13047365; doi:10.12688/wellcomeopenres.25811.1)
Supplement: Peer review history

# Reviewer report: Reviewer response for version 1

**DOI:** 10.21956/wellcomeopenres.28412.r151523

**Published:** 2026-07-16

**Authors:** Annabel Whibley

**Affiliations:** 1 The University of Auckland, Auckland, New Zealand

In this Data Note, Mohan and colleagues report the reference genome assembly of the Golden Plusia, *Polychrysia moneta*under the auspices of Project Psyche. A haplotype-resolved assembly has been generated, with haplotype 1 scaffolded into 31 pseudochromosomes, including the Z sex chromosome. The identity of the specimen was not verified by DNA barcoding. The assembly combines HiFi and Illumina HiC input datasets and follows standard protocols and bioinformatics pipelines for assembly and evalution. Merian element painting was included. The quality of the assembly is excellent, with comparable completeness for both haplotypes (so much so that the red line for hap1 in theFigure 5  kmer profile  appears absent since it is so closely overlaid by the blue line for hap2. There does appear to be a cloud of high coverage, low GC contigs on the BlobPlot- consistent with some kind of contaminant/co-biont) and identification of the source of these would be interesting.

Are sufficient details of methods and materials provided to allow replication by others?

Yes

Is the rationale for creating the dataset(s) clearly described?

Yes

Are the datasets clearly presented in a useable and accessible format?

Yes

Are the protocols appropriate and is the work technically sound?

Yes

Reviewer Expertise:

Bioinformatics, Genomics

I confirm that I have read this submission and believe that I have an appropriate level of expertise to confirm that it is of an acceptable scientific standard.

# Reviewer report: Reviewer response for version 1

**DOI:** 10.21956/wellcomeopenres.28412.r151527

**Published:** 2026-04-02

**Authors:** Sarah Inwood

**Affiliations:** 1 University of Otago, Dunedin, New Zealand

This data note described a chromosome-level genome for *Polychrysia moneta. *This high-quality genome was assembled from PacBio HiFi  and Illumina Hi-C reads from a male specimen, and contains two haplotypes and a mitochondrial genome. This genome has not yet been annotated, but will be in future using RNA-seq data.

 Minor comments:

- The Blob toolkit GC-coverage graph showed a number of short scaffolds with much lower GC content and slightly higher read depth, with their phylum membership having no hit. Did the authors explore what these shorter low-GC content scaffolds are likely to correspond to? 

- Mitofinder was mentioned in text but a version was not provided in the software table. Conversely, bedtools, cooler, fasta_windows, and sanger-tol/curationpretext were all in the software table but not mentioned in text - what were these tools used for?

Are sufficient details of methods and materials provided to allow replication by others?

Yes

Is the rationale for creating the dataset(s) clearly described?

Yes

Are the datasets clearly presented in a useable and accessible format?

Yes

Are the protocols appropriate and is the work technically sound?

Yes

Reviewer Expertise:

Genomics, host-parasite interactions, parasitoid wasps, insects, viruses, biocontrol

I confirm that I have read this submission and believe that I have an appropriate level of expertise to confirm that it is of an acceptable scientific standard.

# Reviewer report: Reviewer response for version 1

**DOI:** 10.21956/wellcomeopenres.28412.r148640

**Published:** 2026-03-12

**Authors:** Melody Clark

**Affiliations:** 1 Natural Environment Research Council, Cambridge, UK

This brief Data Note describes the genome sequence of a male Golden Plusia moth. The assembly is at haplotype level with scaffolds comprising 31 chromosomal pseudochromosomes including the Z sex chrosomome and the mitochondrial genome. The biology and habitat of this species is described, with the sequencing impetus provided by Project Psyche under the auspices of the WSI Tree of Life. DNA extraction and PacBio/Hi-C sequencing were conducted using standard protocols with assembly generated using WSI Tree of Life pipelines with some manual curation. Thus the genome is of very high quality. Chromosome painting has also been performed using Merian elements identifying ancestral linkage groups. Annotation is currently pending on RNA-seq data.

Are sufficient details of methods and materials provided to allow replication by others?

Yes

Is the rationale for creating the dataset(s) clearly described?

Yes

Are the datasets clearly presented in a useable and accessible format?

Yes

Are the protocols appropriate and is the work technically sound?

Yes

Reviewer Expertise:

Transcriptomics and genomics of non-model species

I confirm that I have read this submission and believe that I have an appropriate level of expertise to confirm that it is of an acceptable scientific standard.
